# Does the hyperextension maneuver prevent knee extension loss after arthroscopic anterior cruciate ligament reconstruction?

**DOI:** 10.1007/s10195-016-0408-9

**Published:** 2016-05-10

**Authors:** Hamidreza Yazdi, Amin Moradi, Aida Sanaie, Armin Ghadi

**Affiliations:** 1 Department of Knee Surgery, Firoozgar Hospital, Iran University of Medical Science, Tehran, Iran; 2 Knee Surgery Fellowship, Department of Knee Surgery, Shafa Rehabilitation Hospital, Iran University of Medical Science, Tehran, Iran; 3 Firoozgar Hospital, Iran University of Medical Science, Tehran, Iran

**Keywords:** ACL reconstruction, Extension loss, Hyperextension

## Abstract

**Background:**

Disruption of the anterior cruciate ligament (ACL) is one of the most frequent musculoskeletal injuries affecting physically active men and women. In the United States, an estimated 200,000 ACL reconstructions are performed annually. One of the most common complications of ACL reconstruction is loss of extension. The purpose of this study was to assess the effects of the hyperextension maneuver on preventing knee extension loss after arthroscopic ACL reconstruction.

**Materials and methods:**

In this prospective randomized clinical trial study, 100 adult patients with a documented complete ACL tear were randomized to two groups. All patients underwent arthroscopic ACL reconstruction with quadrupled semitendinosus and gracilis autograft by the senior author based on the same technique and instruments. However, the hyperextension maneuver was only performed in 50 patients during autograft fixation on the tibial side (case group). The postoperative rehabilitation protocol was similar for both groups. The knee range of motion and extension limit was evaluated at 2, 6, 12, and 24 weeks and at 1 year postoperatively.

**Results:**

One hundred patients (88 male and 12 female) aged from 17−36 years (average 26.9 years) were included in our study. The two groups were similar regarding age, sex, and dominant side involvement (*P* >0.4).The difference between the two groups was significant only at 2 weeks (*P* <0.02). After 2 weeks, although the rate of limited extension was higher in the control group, no significant difference was seen between the groups.

**Conclusion:**

Although the hyperextension technique during graft fixation on the tibial side may induce better range of motion in the first 2 weeks after ACL reconstruction surgery, this effect is not significant after 2 weeks.

**Level of evidence:**

Therapeutic level II.

## Introduction

Disruption of the anterior cruciate ligament (ACL) is one of the most frequent musculoskeletal injuries affecting physically active men and women [1]. In the United States, an estimated 200,000 ACL reconstructions (ACLRs) are performed annually, and the incidence of ACL injury is roughly one in 3,000 per year [1–3]. One of the most common complications of ACLR is loss of extension, which is often functionally worse for patients than their preoperative instability [4, 5]. Limited range of motion (ROM) after ACLR has been minimized by improved surgical techniques and perioperative rehabilitation programs [5–7]. Although the amount of initial tension applied to the graft and the position of the knee during application of the tension has a direct effect on stability and ROM of the knee, the precise measurements have not been determined.

The hyperextension technique for hamstring autograft fixation during ACLR was introduced by Pinczewsk and colleagues who recommended a type of hyperextension maneuver on the knee at the time of tibial side graft fixation [8].

The purpose of the study was to assess the effect of the hyperextension maneuver on prevention of knee extension loss after arthroscopic ACLR. The hypothesis was that the hyperextension maneuver can decrease or eliminate knee extension loss after arthroscopic ACLR.

## Materials and methods

This is a prospective randomized clinical trial study conducted between July 2012 and July 2013. The study was approved by the local Medical Ethics Committee.

One hundred adult patients with a documented complete ACL tear were randomized to two groups according to their hospital admission number. All participants gave written informed consent for inclusion in the study. Arthroscopic ACLR was performed for all patients; however, at the time of tibial side graft fixation, the hyperextension maneuver was carried out in 50 patients (case group) and was not performed in the remaining 50 patients (control group). All the patients underwent single-bundle ACLR with quadrupled semitendinosus (ST) and gracilis (G) autograft by the senior author based on the same technique and instruments, except for autograft fixation on the tibial side.

The inclusion criteria were complete ACL tear in adult patients, normal quadriceps force and full ROM of the knee without effusion and edema before surgery (at least 3 weeks after trauma). The exclusion criteria were elderly or skeletally immature patients, multiple ligament injuries, revised ACLR, partial ACL tear, ACLR using allograft, presence of impingement on the intercondylar roof or lateral wall at the time of ACLR, cyclops formation, previous knee surgery (except diagnostic arthroscopy or partial meniscectomy), concomitant meniscal repair or other reconstruction surgery, arthritic changes or grade III–IV chondral damage, limited knee ROM or hyperextended knee preoperatively and uncooperative patients.

All operations were performed by a senior surgeon (HRY) with the same equipment and surgical technique. Preoperative intravenous antibiotics (cephazolin 1 g) were administered approximately 30 min before the incision was made.

After general or spinal anesthesia in the supine position with tourniquet control, preparation and draping was carried out. With one longitudinal incision the ST and G tendons were harvested. Using standard anterolateral and anteromedial portals, the knee was visualized.

The femoral tunnel position was first identified and drilled using a Kirschner wire in an anatomic position through the anteromedial portal with the knee flexed at 110°–120° of flexion (10 o’clock for the right knee and 2 o’clock for left knee).

The tibial tunnel was then prepared in an anatomic position at the ligament footprint using an endoscopic aimer (Karl Storz GmbH, Tuttlingen, Germany) adjusted to a 50° or 55° position in the sagittal plane. In every case, a button (Flipptack; Karl Storz) was used for femoral fixation, and a bioabsorbable screw (MegaFix screw; Karl Storz) was used on the tibial side. Appropriate pretensioning of the graft was performed by cyclic flexion and extension of the knee for 20 repetitions.

In the case group, the graft was fixed using the hyperextension technique. With the knee in its resting position at 20° of flexion, 80 N tension using a tensiometer (Karl Storz) was applied to the four-stranded graft. While maintaining tension on the graft, a bioabsorbable screw was advanced until the screw captured the graft. The knee was then slowly extended up to 5° of hyperextension, allowing any slippage of the bundles to occur. With the knee in extension, and with 80 N tension, the screw was advanced up the tibial tunnel until the bottom was at the level of the tunnel entrance.

In the control group, the graft was fixed at 20° of knee flexion with 80 N tension on the graft without the hyperextension maneuver.

The postoperative rehabilitation protocol was similar for both groups. For the first 3 weeks, walking with crutches was allowed with an extension brace. Weight bearing was allowed as tolerated. Patients were encouraged to restore full extension of the knee and strengthen the quadriceps muscle power as soon as possible. Early knee ROM was performed. Four weeks after surgery, the patients were encouraged to resume daily activities and return to sport was delayed up to 6 months. Patients were followed at 2, 6, 12, 24 weeks and at 1 year postoperatively by another knee surgeon who was not associated with the surgery and was blinded to the surgical procedure. The knee ROM and extension limit was measured using a goniometer and knee radiograph at full passive extension. Limited extension of <3° was considered normal [22]. Severity of extension loss was classified as shown in Table 1.

**Table 1:** Severity of loss of extension of the knee

Loss of extension	Severity score
0–3	Normal
4–5	Mild
6–10	Moderate
>10	Severe

The results were statistically analyzed using SPSS software package for Windows ver. 17.0 (SPSS, Chicago, IL, USA).

A *P* value of <0.05 was considered significant.

## Results

One hundred patients (88 male and 12 female) were included in this study. One patient from the case group and one patient from the control group were excluded from the study due to loss of follow-up. The age of the patients ranged from 17−36 years (average 26.9 years). In 70 % of patients, the dominant leg was involved. The two groups were similar regarding age, sex and dominant side of involvement (*P* > 0.4). The knee ROM and extension loss were evaluated at each examination at 2, 6, 12 and 24 weeks and at 1 year postoperatively. The results are summarized in Table 2.

**Table 2:** Loss of extension in the two groups (with and without the hyperextension maneuver)

Group	Loss of extension (°)	Time after ACL reconstruction			
		2 weeks (%)	6 weeks (%)	12 weeks (%)	24 weeks (%)
Case group (with the hyperextension maneuver)					
Valid	0–3	78	96	98	100
	4–5	18	2	2	0
	6–10	4	2	0	0
	>10	0	0	0	0
	Total (%)	100	100	100	100
Control group (without the hyperextension maneuver)					
Valid	0–3	56	80	92	96
	4–5	38	16	8	4
	6–10	2	4	0	0
	>10	4	0	0	0
	Total (%)	100	100	100	100
*P* value		0.02	0.071	0.172	0.156

**Fig. 1:**
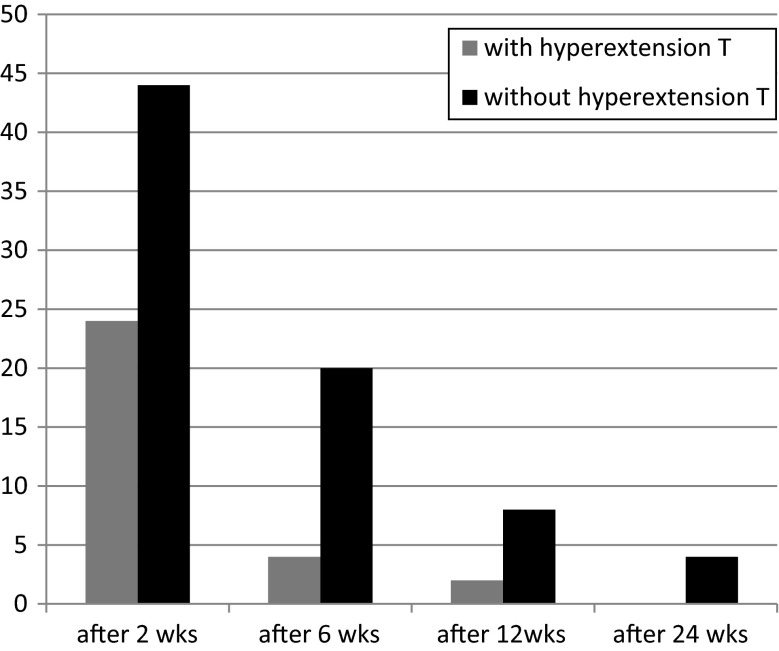
The percentage of patients with loss of extension after arthroscopic ACL reconstruction

The difference between the two groups was significant only at 2 weeks (*P* <0.02). Twenty-two percent of patients in the case group and 44 % of patients in the control group had an extension loss of >3° in week 2. After 2 weeks, although the rate of limited knee extension was higher in the control group, no significant difference was seen between the two groups. After 24 weeks, four patients in the control group suffered from extension loss but in the case group all patients had full ROM in the affected knee. After 1 year all patients had full knee extension. Figure 1 shows the nature of the recovery of knee ROM after ACLR in the case and control groups.

## Discussion

Arthrofibrosis or loss of motion is a known complication after ACLR occurring in 4–35 % of cases [9, 10]. Loss of motion after ACLR causes significant pain and functional impairment [5]. It is identified at the postoperative appointment as loss of full extension (5°–10°) or restricted flexion (120°–125°). Loss of flexion is generally not as disabling as loss of extension [7].

Use of the descriptive term ‘loss of extension’ is preferred to the often misleading terms of ‘arthrofibrosis’ and ‘flexion contracture’ [4]. A loss of extension of >10° prevents a normal gait and increases the load across the patellar femoral joint, resulting in anterior knee pain [11].

To our knowledge this is the first study to evaluate the effect of the hyperextension maneuver on knee extension loss. The results at 2 weeks postoperatively showed a significant difference between the two groups but it was not significant at 6, 12 and 24 weeks. This means that the hyperextension maneuver is effective in preventing knee extension loss shortly after surgery but it is not effective after 2 weeks.

Burks and Leland suggested 3.6 lb of tension for patellar tendon grafts when the knee is at 20–25° of flexion [12]. Bylski-Austrow et al. noted in a cadaveric study that knees tensioned in 30° of flexion are over-constrained and this is independent of the initial tension used [13]. Melby et al. also reported similar results [14].

Nabors et al. evaluated 57 patients after ACLR with a patellar tendon autograft in which the graft was tensioned by a maximal sustained one-handed pull on the tibial end, with the knee in full passive extension. They concluded that tensioning of the graft in full extension ensures that the knee will come to full extension without compromising the stability of the knee [15].

The etiology of limited knee ROM after ACLR is often multifactorial. Elderly, male gender and concomitant ligament reconstructions, especially the medial collateral ligament (MCL), may be considered as risk factors for knee stiffness after ACLR [16, 17]. However, some of the errors are technical errors and depend on the surgeon. The most important risk factor appears to be related to the acuteness of reconstruction [5]. Numerous reports in the literature describe higher rates of knee stiffness when the surgery is performed within 3 weeks of injury; however, some new studies showed that the timing of acute ACLR has less of an effect than originally postulated [4, 6, 18]. Nonanatomic tibial and femoral position, amount of graft tension and position of the knee during graft fixation on tibial side also have an influence on the ROM of the knee after ACLR. Other factors include prolonged immobilization, infection, poor patient compliance, scarring in the intercondylar notch, capsulitis, cyclops lesion and reflex sympathetic dystrophy [16]. Several studies report that early ROM therapy emphasizing immediate postoperative ‘hyperextension’ and avoiding immobilization in flexion reduces the rate of extension loss [4]. It appears that ACLR may be performed with the knee in full extension during graft placement with excellent results and a very low rate of extension loss [16].

In our study, exact exclusion and inclusion criteria were carefully planned in order to exclude factors that may affect the results of our study. All the patients were young with an isolated complete ACL tear and normal knee ROM preoperatively. Concomitant ligament reconstruction especially MCL, and meniscal repair or cartilage reconstructions were considered as exclusion criteria. Anatomic ACLRs in both groups were performed by the same knee surgeon and with same instrumentations and surgical technique. The rehabilitation program was the same in both groups. An extension brace was used in all patients for 3 weeks and early ROM exercise was encouraged.

Over-tensioning ACL grafts may lead to abnormal knee kinematics [19, 20]. In addition the degree of knee extension during graft fixation may affect postoperative motion [9, 22, 23]. Austin et al. in a cadaveric study showed that the level of graft tension (44 N or 89 N) did not affect knee extension; however, tensioning the graft in knee flexion was associated with extension deficits. The authors reported that grafts tensioned and fixed at 30° of flexion had >12° increase in knee flexion after ACLR compared with those tensioned and fixed at full extension [21]. From a two-part biomechanical and clinical study, Nabors et al. suggested that grafts tensioned in full extension result in a low incidence of knee motion loss. In their series of 57 patients who underwent patellar tendon autograft ACLR, only one patient had a mild (5°) extension loss [11].

Harner et al. retrospectively reviewed 244 ACLRs for postoperative stiffness and found an incidence of 11.1 %. Factors associated with loss of motion included acute reconstruction <1 month from injury, male gender, and concomitant MCL repair [16]. In the current study, the extension loss of the knees at 2 weeks after surgery in the case and control groups was 22 and 44 %, respectively; however, the difference was not significant after 2 weeks. The rate of extension loss after 24 weeks was zero in the case group and 4 % in the control group which is less than in the study by Harner et al. There was no significant gender difference between the two groups. All the patients underwent isolated ACLR at least 4 weeks after initial trauma.

Some surgeons believe that delay in full extension exercises may protect the reconstructed ACL. Otto et al. retrospectively reviewed the 5-year results of 68 patients who underwent single-bundle ACLR. Extension loss of >3° was seen in 5 % of the patients; however, the postoperative therapy regimen consisted of the use of a brace which did not allow full extension for the first 4 weeks after reconstruction. It was concluded that this technique results in excellent stability of the knee and allows return to a high level of function [22]. On the other hand, in an analysis of data from 191 consecutive patients with ACLR, 12 % developed arthrofibrosis [17]. This study showed that postoperative limitation in knee extension may increase the risk of extension loss. Bracing the knee in full extension with motion starting within 24 h dropped the incidence of arthrofibrosis from 23 to 3 % [11]. All patients in the current study used an extension brace up to 3 weeks just for walking and early knee ROM exercises especially extension exercises was encouraged. Therefore, the effect of postoperative rehabilitation was excluded.

One main concern is that full active and passive extension immediately after ACLR may increase postoperative laxity of the knee. Isberg et al. during a randomized and prospective study with a 2-year follow-up showed that a postoperative rehabilitation protocol including active and passive extension without any restrictions in extension immediately after an ACLR did not increase the postoperative anterior–posterior knee laxity [23]. In the current study the patients in both groups were allowed to do full active and passive knee extension immediately after drainage removal. As the postoperative protocols of the two groups in our study were the same, the factors that may affect the ROM of the knee and results were excluded.

A limitation of our study was that the effect of the hyperextension maneuver on graft laxity after ACLR surgery was not evaluated and needs further study.
